# Using Fracture Patterns and Planned Operative Modality to Identify Fractured Neck of Femur Patients at High Risk of Blood Transfusion

**DOI:** 10.7759/cureus.18220

**Published:** 2021-09-23

**Authors:** Benjamin Gowers, Michael S Greenhalgh, Olivia J McCabe-Robinson, Chea Tze Ong, Joseph E McKay, Kathryn Dyson, Karthikeyan P Iyengar

**Affiliations:** 1 Trauma and Orthopaedics, Kettering General Hospital, Kettering, GBR; 2 Trauma and Orthopaedics, Health Education England North West, Manchester, GBR; 3 Orthopaedics, Health Education England North West, Manchester, GBR; 4 Trauma and Orthopaedics, National Health Service (NHS) Education for Scotland, Edinburgh, GBR; 5 Trauma and Orthopaedics, Southport and Ormskirk Hospital National Health Service (NHS) Trust, Southport, GBR

**Keywords:** outcomes, blood transfusion, anaemia, femoral neck fracture, hip fracture

## Abstract

Background

Fractured neck of femurs is common, serious injuries usually requiring operative management. Red blood cell transfusions are often required to treat perioperative anaemia, but these are not without adverse effects.

Aims and objectives

The aim of this study is to identify subgroups of fractured neck of femur patients more likely to require red blood cell transfusions. We try to identify targeted strategies to reduce blood transfusion-associated adverse effects and thus improve outcomes.

Design and methods

A retrospective cohort study of 324 patients. Patients were divided into cohorts based on radiological fracture patterns and operations performed. Data were collected from patient records, picture archiving and communication systems, the local transfusion laboratory, and the national hip fracture database. The primary outcome was blood transfusion rates in different fracture patterns in fractured necks of femur patients. The secondary outcome was blood transfusion rates in different operation types for fractured neck of femur patients. Chi-squared tests for independence were performed.

Results

14.9%, 34.7% and 33.3% of patients with intracapsular, intertrochanteric and subtrochanteric fractures, respectively, received blood transfusions. There was a significant relationship between fracture pattern and blood transfusion (*X*^2^ (2, N = 324) = 17.1687, p = 0.000187).

47% of patients receiving long intramedullary nails, 45% of short intramedullary nails, 27% of open reduction internal fixations, 18% of hemiarthroplasties and 9% of total hip arthroplasties resulted in blood transfusions. There was a significant relationship between operative modality and blood transfusion (*X*^2^ (4, N = 302) = 22.0184, p = 0.000199).

Conclusion

In patients who have sustained a fractured neck of the femur, the fracture pattern and operative modality are both independently associated with the rates of red blood cell transfusion. In these identified groups, we propose that increased vigilance and awareness regarding transfusion avoiding strategies are utilised with the goal of improving patient outcomes.

## Introduction

A fracture to the neck of the femur (NOF) is a serious and common injury. According to the latest report from the National Hip Fracture Database (NHFD) 67,302 patients with NOF fractures presented to hospitals in the United Kingdom in 2019 [1]. Due to the high mortality rates of 10% at 30 days and 30% at one year post-injury according to the National Institute for Health and Care Excellence (NICE) [1], a full Multidisciplinary Team (MDT) approach is paramount. Input from orthogeriatricians, orthopaedic surgeons, anaesthetists, nursing, and allied health professionals with expertise appropriate for these frail patients facilitates preoperative optimisation and improves outcomes [2]. Guidance from the British Orthopaedic Association (BOA) and NICE recommends early surgical intervention occurs in a timely manner, as this has been shown to improve clinical outcomes [1-3]. To help facilitate best practice for fractured NOF patients and to improve outcomes, a Best Practice Tariff (BPT) is paid to hospitals that meet targets related to MDT working and timely surgery [2].

In fragility hip fractures, the Nottingham Hip Fracture score is used to assess the risk of mortality. One of the independent factors shown to be associated with increased mortality is low haemoglobin (Hb) on admission. This is pertinent as most fractured NOF patients require surgical intervention as part of their management [4,5]. However, low admission Hb alone has not shown to be a good predictor of peri-operative blood transfusion requirement [6].

Anaemia can be managed by red blood cell (RBC) transfusion once a threshold of Hb is reached. There are accepted guidelines for initiation of RBC transfusion whereby the Hb drops below 70g/litre, except in the case of concurrent coronary artery disease where the threshold is a Hb below 80g/litre. In clinically symptomatic patients, a single unit of RBC can be transfused for a Hb below these thresholds, with a repeat check of Hb levels post-transfusion. If further transfusions are required to reach the target concentration of 70-90g/litre or 80-100g/litre for patients with coronary artery disease, then Hb checks need to occur between transfusions [7,8].

Although effective in treating anaemias, allogeneic RBC transfusions are known to have acute serious adverse effects, these include febrile, anaphylactic, and haemolytic transfusion reactions, transfusion-related acute lung injury (TRALI), and transfusion-associated circulatory overload (TACO) [7,9]. In addition to these adverse effects, Arshi et al described a significantly higher mortality rate at 30 days post-injury (OR 1.29, p < 0.05) and increased length of hospital stay (p < 0.05) if an RBC transfusion was received by fractured NOF patients [10]. This correlates with another study from the United States indicating that blood transfusions were independently associated with length of stay and mortality [11].

There are other options available for patients who are expected to have significant blood loss during surgery or are anticipated to require a blood transfusion peri-operatively. One of these is Intra-operative Cell Salvage (ICS). This involves collecting the patient’s own blood from the surgical field, using a centrifuge to separate out RBCs and reinfusing them. Indications for ICS include an expected blood loss of over one-fifth of a patient’s blood volume and patient factors restricting allogeneic RBC transfusion, such as in the case of Jehovah’s Witness patients [9]. The UK Cell Salvage Action Group (UKCSAG) also suggests that ICS should be considered for patients undergoing elective or emergency surgical procedures who have risk factors for bleeding or low pre-operative Hb levels [12].

A Cochrane review assessing the efficacy of ICS in reducing allogeneic RBC transfusion and the evidence for any effect on clinical outcomes found that ICS was effective at reducing the need for RBC transfusions in adult orthopaedic surgery. It also found that the use of cell salvage did not appear to impact adversely on clinical outcomes [13].

Other strategies for patient optimization to reduce RBC blood transfusion risk in patients having surgery include oral and intravenous (if not enough time for oral iron to be effective) iron supplementation and erythropoietin. Intra-operative strategies include the use of regional anaesthesia, maintaining normothermia, use of tranexamic acid and point of care testing (POCT) to give rapid information regarding a patient’s Hb level [8,12].

The aim of our study is to identify subgroups of patients within the cohort of fractured NOF patients who are more likely to require a blood transfusion. Once high-risk patients are identified, our goal is to identify targeted strategies to reduce blood transfusion-associated adverse effects and thus improve outcomes.

## Materials and methods

Study population

A retrospective cohort study of 324 consecutive NOF fracture patients, who presented to a district general hospital in the United Kingdom over a one-year period, between October 1, 2017 and September 30, 2018. All patients who presented with a fractured NOF were included. None of the patients who presented were excluded from the study.

Study cohorts

**Figure 1:**
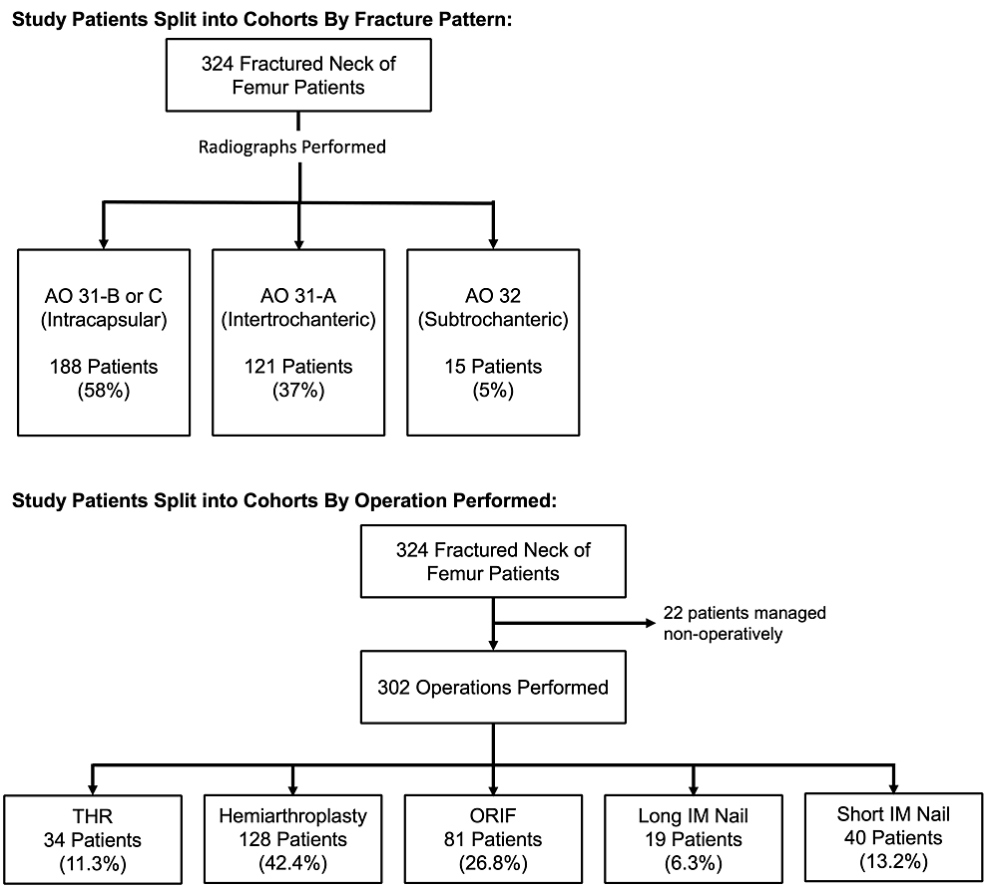
Study patients split into cohorts by fracture pattern and operation performed Abbreviations: AO = AO/OTA Fracture and Dislocation Classification Compendium; THR = Total Hip Replacement/Arthroplasty; Hemiarthroplasty = Hip Hemiarthroplasty; ORIF = Open Reduction Internal Fixation; Long IM Nail = Long Intramedullary Nailing; Short IM Nail = Short Intramedullary Nailing

All patients had anteroposterior radiographs taken of their pelvis and lateral hip on their affected side as part of their initial triage on admission to the emergency department (ED). Patients were then divided into cohorts based on the radiological pattern of their fracture according to the AO/OTA fracture and dislocation classification [14]. The cohorts were AO 31-B or C (intracapsular), AO 31-A (intertrochanteric) and AO 32 (subtrochanteric) fractures (Figure 1). The primary outcome measure was the rate of RBC transfusion in each cohort. The secondary outcome measure was the mortality rate at one year post-injury.

Separately, patients were divided into five cohorts dependent on their subsequent type of operation performed. Twenty-two patients did not receive operative management and were excluded. The cohorts were patients undergoing total hip replacement/arthroplasty (THR), hip hemiarthroplasty, long intramedullary nailing and short intramedullary nailing. Only six patients underwent an operation with cannulated hip screws, which was deemed too few for statistical analysis. These patients were therefore included with those who underwent a dynamic hip screw operation, the operation deemed to be most similar by the authors, to form a cohort with the label closed or open reduction and internal fixation (ORIF) (Figure 1). The primary outcome measure was the rate of RBC transfusion in each cohort.

Data sources and collection

Data for this study were collected from patient records, Electronic Patient Records (EPR), Picture Archiving and Communication System (PACS), local transfusion laboratory records and the National Hip Fracture Database (NHFD). Patient demographics, patient’s pre-injury residence status, pre-operative American Society of Anaesthesiologists Physical Status Classification System (ASA Grade), AO/OTA fracture classification, an operation performed, peri-operative blood transfusion requirements and mortality at one year post-injury were recorded.

Statistical analysis

Statistical analysis of data was with Statistical Package for the Social Sciences software (SPSS for mac, build 1.0.0.1461, IBM). Mean and standard deviations were used to summarise the data for continuous variables and frequency/percentage with standard deviations (SD) for categorical variables. Chi-squared tests for independence were performed, to determine if any relationship between fracture pattern and transfusion rates, or operation performed and transfusion rates were due to chance or relationship between the variables. A p-value of less than 0.05 was considered significant.

Patient consent and study ethical review

The patients gave their written informed consent; for patients who lacked capacity, consent was obtained in accordance with the local consenting and ethics guidelines and discussions with their families. The review board of the local research and clinical effectiveness department approved the study design and gave ethical approval (Local Study ID 19-278).

## Results

Demographics

Three hundred twenty-four patients presented with a fractured NOF, all of whom were included in the study. Mean age was 88.2 (SD 7.7), 77.4% (n = 251) were female and 22.5% (n = 73) were male. The mean ASA grade was 2.8 (SD 0.62).

Patient demographics split into cohorts are shown in Table 1.

**Table 1:** Demographics, ASA grade and pre-injury residence status of patients, grouped by fracture pattern in the study Abbreviations: ASA = American Society of Anaesthesiologists Physical Status Classification System; AO = AO/OTA Fracture and Dislocation Classification SD = Standard Deviation; M = Male; F = Female

Parameters	Intracapsular Fracture (AO 31-B or C) (n = 188)	Intertrochanteric Fracture (AO 31-A) (n = 121)	Subtrochanteric Fracture (AO 32) (n = 15)
Demographics			
Age (Years) (Mean, SD)	82.5 (7.52)	84.4 (7.94)	83.3 (7.00)
Gender (M : F, % female)	42 : 146, (77.7%)	24 : 97, (80.2%)	7 : 8, (53.3%)
ASA Grade n, (% of cohort)			
1	2, (1.1%)	1, (0.8%)	0
2	46, (24.5%)	29, (24.0%)	2, (13.3%)
3	126, (67.0%)	76, (62.8%)	11, (73.3%)
4	14, (7.4%)	14, (11.6%)	2, (13.3%)
5	0	1, (0.8%)	0
Residence Pre-Injury n, (% of cohort)			
Own Home	142, (75.5%)	89, (73.6%)	10, (66.6%)
Residential Home	29, (15.4%)	21, (17.4%)	3, (20%)
Nursing Home	17, (9.0%)	11, (9%)	2, (13.3%)

Radiological fracture pattern classification and transfusion rates

The fractured NOFs were classified as 188 (58%) AO 31-B or C (intracapsular), 121 (37%) as AO 31-A (intertrochanteric) and 15 (5%) as AO 32 (subtrochanteric) fractures.

14.9% (n = 28) of intracapsular, 34.7% (n = 42) of intertrochanteric and 33.3% (n = 5) of subtrochanteric fractures received transfusions (Table 2). There was a significant relationship between fracture pattern and blood transfusion (X^2^ (2, N = 324) = 17.1687, p = 0.000187).

**Table 2:** RBC transfusion rates and one-year mortality of patients grouped by fracture pattern in the study Abbreviations: AO = AO/OTA Fracture and Dislocation Classification; RBC = Red Blood Cell

Parameters	Intracapsular Fracture (AO 31-B or C) (n = 188)	Intertrochanteric Fracture (AO 31-A) (n = 121)	Subtrochanteric Fracture (AO 32) (n = 15)
RBC Transfusions			
Received RBC Transfusion	28	42	5
No Transfusion	160	79	10
Percentage Transfused	14.9%	34.7%	33.3%
One-Year Mortality			
Alive	124	77	8
Deceased	64	44	7
Percentage One-Year Mortality	34.0%	36.4%	46.6%

Operative management and transfusion rates

Three hundred two operations were performed, of which 128 patients underwent hip hemiarthroplasties, 81 had ORIF of their fractures, 40 patients had short intramedullary nailing and 19 long intramedullary nailings. Thirty-four patients received THRs for intracapsular fractures following NICE CG 124 criteria [1]. No operation was performed in 22 patients.

**Figure 2:**
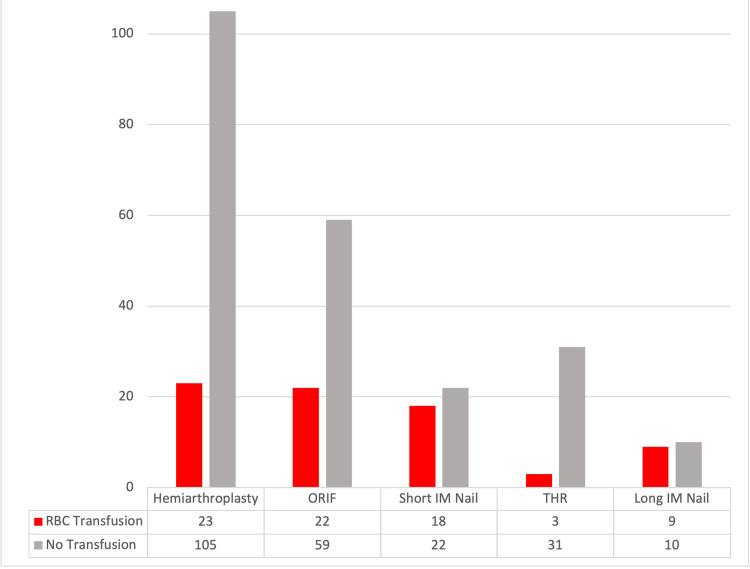
Transfusion rates in fractured neck of femur patients grouped by operation type Abbreviations: RBC = Red Blood Cell; Hemiarthroplasty = Hip Hemiarthroplasty; ORIF = Open Reduction Internal Fixation; IM = Intramedullary; THR = Total Hip Replacement

47% (n = 9) of long intramedullary nails, 45% (n = 18) of short intramedullary nails, 27% (n = 22) of ORIFs, 18% (n = 23) of hemiarthroplasties and 9% (n = 3) of total hip arthroplasties resulted in blood transfusions (Figure 2). There was a significant relationship between operative modality and blood transfusion (X^2^ (4, N = 302) = 22.0184, p = 0.000199).

Mortality

At one year post injury, 34% (n = 64) of intracapsular fractures, 36.4% (n = 44) of intertrochanteric fractures and 46.6% (n = 7) of subtrochanteric fractures were deceased (Table 2).

At one year post injury, 36.7% (n = 47) of hemiarthroplasty patients, 30.9% (n = 25) of ORIF patients, 32.5% (n = 13) of short IM nail patients, 8.8% (n = 3) of THR patients and 63.2% (n = 12) of long IM Nail patients were deceased (Table 3).

**Table 3:** RBC transfusion rates and one-year mortality grouped by operation type Abbreviations: Hemiarthroplasty = Hip Hemiarthroplasty; ORIF = Open/Closed Reduction and Internal Fixation; IM = Intramedullary; THR = Total Hip Replacement/Arthroplasty; RBC = Red Blood Cell;

Parameters	Hemiarthroplasty (n = 128)	ORIF (n = 81)	Short IM Nail (n = 40)	THR (n = 34)	Long IM Nail (n = 19)
RBC Transfusions					
Received RBC Transfusion	23	22	18	3	9
No Transfusion	105	59	22	31	10
Percentage Transfused					
One-Year Mortality					
Alive	81	56	27	31	7
Deceased	47	25	13	3	12
Percentage One-Year Mortality	36.7%	30.9%	32.5%	8.8%	63.2%

## Discussion

Bleeding from the skeletal and periosteal vasculature, damage to surrounding vascular and soft tissue structures, and the inflammatory phase of bone healing all contribute to blood loss and a drop in intravascular Hb [15]. Operative management is associated with a further risk of bleeding. The combination of physiological blood loss due to injury and operative intervention thus contributes to Hb depletion and the requirement for transfusion.

Fractured NOF is an umbrella term that includes all fractures of the proximal femur; however, their management strategies differ. In the UK, even diaphyseal subtrochanteric femoral fractures are included in this group for inclusion in the NHFD [2]. Fracture patterns and patient factors, such as cognition and mobility status, influence these differences. The blood supply to the femoral head is largely supplied by the medial and lateral circumflex femoral arteries, branches of the profunda femoris artery. These arteries anastomose at the base of the femoral neck to form a ring, from which smaller vessels arise and run proximally to supply the hip joint itself [16]. Surgical planning of which operation to perform is a multifactorial decision. This includes fracture pattern stability, for example, an oblique intertrochanteric fracture may be treated with a Dynamic (Sliding) Hip Screw (DHS), whereas a reverse oblique pattern requires intramedullary fixation. Threat to the blood supply of the femoral head must also be considered as if this is at risk the femoral head itself must be replaced via arthroplasty. Other factors that can influence the type of operation performed, for example, to decide between a hip hemiarthroplasty and a THR, include a patient's comorbidities and baseline functional status. To help inform and guide the best surgical options, classification systems have been developed such as the AO/OTA Fracture Classification [14]. In the management of fractured NOF, especially in the elderly, early rehabilitation is crucial for a satisfactory functional outcome. One of the key factors influencing post-operative mobilisation is anaemia. One of the interventions used to treat anaemia is RBC transfusion [17]. An admission Hb of less than 100g/L is an independent risk factor for mortality for a patient with a fractured NOF, however low admission Hb has not shown to be an accurate predictor of transfusion requirement in their admission [5,6].

In this study, out of a total of 324 patients, 58% sustained intracapsular fractures, 37% intertrochanteric fractures and 5% subtrochanteric fractures. 23% (n = 75) of all patients who sustained a fractured NOF received a RBC transfusion. Divided by fracture pattern, 15% of intracapsular, 35% of intertrochanteric and 33% of subtrochanteric fractures required transfusion. A significant relationship between fracture pattern and transfusion rate was shown with a chi-square test of independence (X^2^ (2, N = 324) = 17.1687, p = 0.000187). These results are consistent with Arshi et al*.* who found that subtrochanteric femoral fractures and intertrochanteric femoral fractures were independent risk factors for receiving blood transfusion [10].

This study found that fractures with intertrochanteric or subtrochanteric patterns were associated with greater than double the transfusion rates of intracapsular fracture patterns. There are several possible explanations for this. Extracapsular fractures result in greater involvement of cancellous bone and greater local soft-tissue injury due to fracture fragments, leading to higher blood loss. With intracapsular fractures, the bony injury is proximal to the anastomotic ring that provides most of the blood supply to the femoral head and the joint capsule itself serves to contain the bleeding by producing a tamponade effect. Consequently, the blood loss in intracapsular NOF fractures is lower than intertrochanteric or subtrochanteric patterns.

One-year mortality was comparable for intracapsular and intertrochanteric fractures, at 34.0% and 36.4%, respectively, but was higher in subtrochanteric fractures at 46.7%. Patients who sustained a subtrochanteric fracture also had the highest rates of RBC transfusion (Table 2). Receiving an RBC transfusion at any point during an acute admission following a fractured NOF carries an increased risk of mortality [6]. The higher mortality and transfusion rates in the subtrochanteric group may reflect greater bony and soft tissue damage due to the injury, with greater blood loss.

Most fractured NOF patients receive operative management for their injury. In this group, hip hemiarthroplasties were most commonly performed, making up 42.4% of all fractured NOF operations (n = 128). Following this, ORIF with either DHS or Cannulated Hip Screws (CHS) was the next procedure most commonly performed (26.8% of operations, n = 81). Long intramedullary nailings were least frequently performed, at 6.3% (n = 19) (Figure 1).

The one-year mortality varied greatly depending on the operation performed. The lowest mortality was observed in the THR group at 8.8% (n = 3), which is perhaps unsurprising given this procedure is offered to patients with the fewest comorbidities and best pre-injury functional baseline. The one year mortality rate for hemiarthroplasty, short IM nail and ORIF were comparable to the expected rate of one third for all NOF fractures suggested by NICE at 36.7% (n = 47), 32.5% (n = 13) and 30.7% (n = 25), respectively [1]. Long IM nails carried a higher rate of one-year mortality at 63.2% (n = 12), again this may be due to greater bony and soft tissue damage, but could also be as a result of more patients in this cohort receiving an RBC transfusion with their associated increased mortality risks [6].

The type of operation performed also affected the rate of RBC transfusion. We have shown that there was a significant relationship between operative modality and blood transfusion with a chi-square test of independence (X2 (4, N = 302) = 22.0184, p = 0.000199). Intramedullary fixation was associated with higher transfusion rates than hip arthroplasty. Notably, intramedullary fixation for proximal femoral fractures is most commonly performed for subtrochanteric fractures. It is unclear whether the extent of tissue damage, as previously discussed, or the use of an intramedullary nail is the predominant factor in the increased transfusion requirement. Whilst these operations can often be performed through a smaller incision in the skin than other operations for fractured NOF, the medullary canal must be widened through reaming to permit passage of the nail. An unavoidable consequence of this process is damage to the intramedullary arterial and venous systems, most notably the nutrient artery system, which serves as the principal blood supply to the bone [18]. Reaming can be a potentially significant source of bleeding and, therefore, may constitute an increased risk of transfusion requirement.

Almost half of the patients undergoing intramedullary nailing received transfusions. Transfusion rates were slightly greater in the group undergoing long intramedullary nailing (47%) versus short intramedullary nailing (45%). Long nails are associated with longer operative time and greater intra-operative blood loss than short nails and the finding of greater transfusion rates in patients undergoing long nailing in our group is consistent with the findings of Boone et al., who compared outcomes of long and short nails in 194 patients treated for proximal femoral fracture with intramedullary nailing [19]. In our study, 8% of hemiarthroplasties and 9% of total hip arthroplasties resulted in blood transfusions. Whilst both of these operations require widening of the proximal part of the femur to allow room for the prosthesis, reaming of the majority of the femur is not required. This, and the fact that arthroplasty is commonly reserved for intracapsular fractures which are associated with lower bleeding risk, as previously described, may offer an explanation of the lower transfusion rates in these groups.

Anaemia in elderly surgical patients can have serious implications, and RBC transfusions are a valuable intervention in its management [16]. However, they are not without risks and adverse effects, which can be compounded by the comorbidities often concurrent in fractured NOF patients [10].

Study limitations

Data for this study were collected retrospectively, reducing the robustness of our observations. Our data was collected from one institution. Neither grade of operating surgeon, transfusion reactions or admission haemoglobin was recorded.

In our future work, we plan for a collaborative multi-centre study, with the inclusion of these additional variables.

## Conclusions

Both fracture pattern and operative modality are independently associated with the rates of RBC transfusion in fractured NOF patients. Patients who are at the highest risk of RBC transfusion are those who sustain a subtrochanteric or intertrochanteric fracture or require a long or short intramedullary nail.

RBC transfusions have been shown to increase morbidity, mortality and length of hospital stay. In these high-risk groups, we propose that additional strategies such as intravenous iron therapy, intra-operative cell salvage and point of care testing should be investigated as a possible means of improving outcomes.
